# The Teaching Pattern of Law Majors Using Artificial Intelligence and Deep Neural Network Under Educational Psychology

**DOI:** 10.3389/fpsyg.2021.711520

**Published:** 2021-10-29

**Authors:** Di Xuan, Delong Zhu, Wenhai Xu

**Affiliations:** ^1^Shi Liang School of Law, Changzhou University, Changzhou, China; ^2^KoGuan School of Law, Shanghai Jiao Tong University, Shanghai, China; ^3^School of Management Engineering, Anhui Institute of Information Technology, Wuhu, China; ^4^School of Law, Tongji University, Shanghai, China

**Keywords:** educational psychology, artificial intelligence, deep learning, teaching design, law majors

## Abstract

With the increasing attention to the cultivation of legal talents, a new teaching model has been explored through artificial intelligence (AI) technology under educational psychology, which focuses on improving learning initiative, teaching methods, and teaching quality of students. First, the application of AI and deep neural network (DNN) algorithms are reviewed in education, and the advantages and disadvantages of traditional learning material recommendation algorithms are summarized. Then, a personalized learning material recommendation algorithm is put forward based on DNN, together with an adaptive learning system based on DNN. Finally, the traditional user-based collaborative filtering (UserCF) model and lifelong topic modeling (LTM) algorithm are introduced as the control group to verify the performance of the proposed recommendation system. The results show that the best learning rate of model training is 0.0001, the best dropout value is 0.5, and the best batch size is 32. The proposed personalized learning resource recommendation method based on deep learning (DL) still has good stability under various training data scales. The personalized test questions of recommended students are moderately difficult. It is easier to recommend materials according to the acquisition of knowledge points and the practicability of the recommended test questions of students. Personalized learning material recommendation algorithm based on AI can timely feedback needs of students, thereby improving the effect of classroom teaching. Using the combination of AI and DL algorithms in teaching design, students can complete targeted personalized learning assignments, which is of great significance to cultivate high-level legal professionals.

## Introduction

Since the 19th National Congress of the Communist Party of China (CPC), the concept of governing a country by law is widely spread, and the demand for legal personnel is urgent. Legal personnel is urgently needed in both academic research and legal practice (Emery and Anderman, 2020). The state advocates the concept of “Internet + education” and points out the strategic direction and specific path for education reform in China. This is a new concept that is different from the traditional teaching concept. It is an innovative teaching model that integrates modern science and technology into education and is designed for large-scale personalized teaching. Artificial intelligence (AI) greatly changes the development mode of various industries. In particular, with the help of “AI +,” universities are exploring the application of AI to the cultivation of talents in mathematics, management, psychology, art, and other disciplines (Haryati, 2018; Hilpert and Marchand, 2018).

Deep learning (DL) is a new research direction of machine learning (ML), and it is considered to be one of the means to realize AI. In this study, the DL algorithm is applied to the field of law education to improve the learning efficiency of law majors. At present, the ability of college students to learn, analyze, and use resources varies greatly, and their desire for knowledge varies greatly. DL begins to be applied to explore the personalized consultation and an online learning platform with massive data. Therefore, the personalized learning resources are combined with DL to cultivate law majors. In addition, the learning experience and efficiency of students are improved based on the new teaching mode (Knox, 2020). At present, the “teacher-centered” teaching model is not suitable for law majors in colleges and universities, who should be proficient in legal skills and theories. Based on the changes in psychology and educational psychology of students, the “student-centered” educational concept is formed, and it innovates the traditional teaching mode, and helps cultivate legal talents with comprehensive quality (Nagao, 2019).

According to the abovementioned data, a personalized learning resource recommendation system based on the deep neural network (DNN) is proposed. The system can predict and recommend the required learning materials based on the learning history of students and their behavior to help them complete the learning tasks. Then, an experiment is designed to verify the performance of the system and analyze the application of AI and DNN. After the traditional learning resource recommendation algorithm is reviewed, a personalized learning resource recommendation system based on DNN is proposed. It aims to help students improve their learning effect through intelligent and targeted learning.

## Literature Review

### Research Status of Related Fields

Many scholars begin to explore AI + education patterns deeply with its continuous advancement, which is of great significance to the innovation of education patterns. Edwards and Cheok (2018) proposed that the shortage of teachers would affect the future of education, and solving the problem of role of teachers could effectively solve some educational problems. Thereby, they introduced AI into the field of education and completed the design of independent robots through teaching delivery. The robots could interact with students and explain relevant knowledge. The results showed that the teacher robot could alleviate the teacher shortage, and the future research direction was prospected (Edwards and Cheok, 2018). Hinojo-Lucena et al. (2019) pointed out the further development of AI in the field of higher education. They found that although there were many studies on AI and education, the research was still in its infancy, and its application in colleges had not been consolidated. Therefore, in future research, it was essential to expand the research scope to truly realize the integration of AI and education (Hinojo-Lucena et al., 2019). Shin (2021) analyzed the definition and attributes of AI thinking for AI-assisted education to facilitate educators to apply AI in education. Based on text mining crawling and co-word analysis, AI thinking was designed and defined using Python programming language. According to the results of co-word analysis, AI thinking adopted a comprehensive thinking process to solve decision-making problems through the process of discussion, provision, demonstration, and certification (Shin, 2021). Lee and Lee (2021) argued that AI was gradually affecting all aspects of daily life, including education. AI could provide special support for learners through academic sustainability or suspension prediction. Through the application of AI in physical education (PE), the applicational potential of AI was expanded, and the teaching method of PE was innovated with visualization function and repeatability. Then, based on the related concept of AI, the principle and application of AI in sports were explored, focusing on the AI applicable sports technology fields, i.e., customized PE courses, knowledge provision, learner evaluation, and learner consultation methods. The results emphasized the professional knowledge that future PE teachers needed in the application of AI. It highlighted the correlation between AI applications and sports technologies (Lee and Lee, 2021). Çağl et al. (2021) tested the confidence of college dental students in anesthesia teaching and research through AI, reflecting the auxiliary role of AI in education. Based on education, Thomas (2021) used AI technology to study the impact of education on poverty and emphasized the implicit effect of education. Hannah et al. (2021) studied the application of AI in pathology and highlighted the contribution of AI in advanced fields. Crystal and Sotiria (2021) studied the application of AI in Finnish teacher education. Moreover, there are many studies related to teaching design and classroom interaction.

### Related Work Analysis

Based on the above analysis of the application of AI in the field of education, the following three important points can be summarized:

First, AI brings many new tools to the experts and scholars in the field of educational research. It is widely used in the design of classroom teaching, which has broad application prospects in this field. It is foreseeable that the development of AI can greatly promote education to informationization and educational reform.

Second, AI is applied in various fields of education due to its powerful function and adaptability, which can promote the innovation and intelligence of talent training in colleges and universities, for the students in majors, such as PE, esthetic education, and mental health education.

Third, legal education is rigorous, logical, and individualized. However, the application of AI to legal education is rare. Therefore, AI and DNN are applied to cultivate the law talents, which provides new ideas for the reform of law education based on human-computer interaction (HCI) technology and DL to improve the interest of students in law learning and their teaching effect.

## Methods and Experiments

This research involves the application of many DL algorithms and the related theories of educational psychology, which are introduced in this section.

### Relevant Theories of Pedagogy

The adaptive learning system focuses on the differences of learners and provides adaptive learning resources, which can overcome the limitations of traditional education and achieve a good learning effect (Mavezera et al., 2019). The design and development of an excellent adaptive learning system should be guided by relevant educational theories and appropriate resource recommendation methods. Based on the above analysis, the relevant theories and recommendation algorithms involved in the adaptive learning system are introduced, the technology needed to implement the system is analyzed, and the personalized learning theory, the target classification theory, and the project response theory are displayed.

Personalized learning is an adaptive learning method according to the ability of students to achieve a clear goal and improve the performance of students efficiently. Although the concept of personalized learning attracts the attention of many countries and scholars, there is no unified and accurate conclusion on the specific definition of personalized learning. At present, the concept of personalized learning recognized in academia can be summarized as follows: (1) personalized learning can provide all students with comprehensive and diverse learning methods; (2) personalized learning is an adaptive arrangement of learning methods and progress based on the interest and experience of learners (Castilla et al., 2018); and (3) personalized learning is a learning method that respects individual learners and fully considers their needs, environments, and thinking. Due to various limitations, traditional personalized learning only relies on the guidance of teachers, and it has many disadvantages. For example, it is dependent on the personalized teaching of teachers, and the requirements for professionalism, experience, and ability of teachers are high (Liu et al., 2018). In addition, learning guidance based on experience cannot be explained scientifically, and it is difficult to spread widely. Under national conditions in China, a teacher often needs to guide students, and it is impossible to get complete fairness. In addition, the traditional teaching methods are difficult to achieve real personalized and efficient learning. With the development of big data and DL, some personalized learning models reveal the hidden relationship between data and the constructed model by analyzing learning data, which plays an important role in analyzing the learning environment of students and providing guidance for personalized learning (Zhang et al., 2019).

Learning goal classification is a framework of theoretical learning. It helps teachers to master the abilities of students, know about the cognitive abilities of students, and make their teaching activities, so it has an important value in the field of education. The project response theory overcomes the relativity in classical measurement theory. If the subject has potential traits, it will affect his/her performance in the test, and then, the permanent project parameters can be established by the project response theory. The research on the response theory is mainly divided into dynamic and static research (Al Mahdi et al., 2019; Liang, 2019). Specifically, the dynamic model of the project response theory is used to analyze the change process of potential characteristics of subjects, and the static model is used to analyze the potential characteristics of subjects at a certain time. However, theoretical research is shallow, and the commonly used model based on the project response theory is static because it starts late.

### Introduction to Basic Recommended Algorithms

With the rapid development of computer technology and Internet technology, diversified and rich learning resources are emerging. Scientific and reasonable selection of these learning resources for students is a problem worth thinking about in the field of AI education. Based on adaptive learning theory, this study attempts to realize targeted learning resource recommendations through AI algorithms. However, first, it is necessary to analyze the traditional basic recommendation algorithms and summarize their advantages and disadvantages. Recommendation algorithm is common, which infers potential user-preference items from their use records using mathematical algorithms, thereby realizing information retrieval and information filtering, and it plays a particularly important role in the present information explosion. Common recommendation algorithms include the following (Ardito et al., 2019; Wang et al., 2020): (1) popularity-based recommendation algorithm, (2) collaborative filtering recommendation algorithm, (3) content-based recommendation algorithm, (4) model-based recommendation algorithm, and (5) hybrid recommendation algorithm.

Thus, five common recommendation algorithms are reviewed, and each has its advantages and disadvantages. The popularity-based recommendation algorithm is simple, without a cold start problem but cannot make personalized recommendations. The collaborative filtering recommendation algorithm is simple and has high recommendation accuracy. The content-based recommendation algorithm has no cold start problem but the recommended content is relatively single. The model-based recommendation algorithm has fast and accurate recommendations and high recommendation efficiency, but the model needs to be maintained frequently (Kai et al., 2019).

### Recommendation Algorithm Based on Deep Learning

**FIGURE 1:**
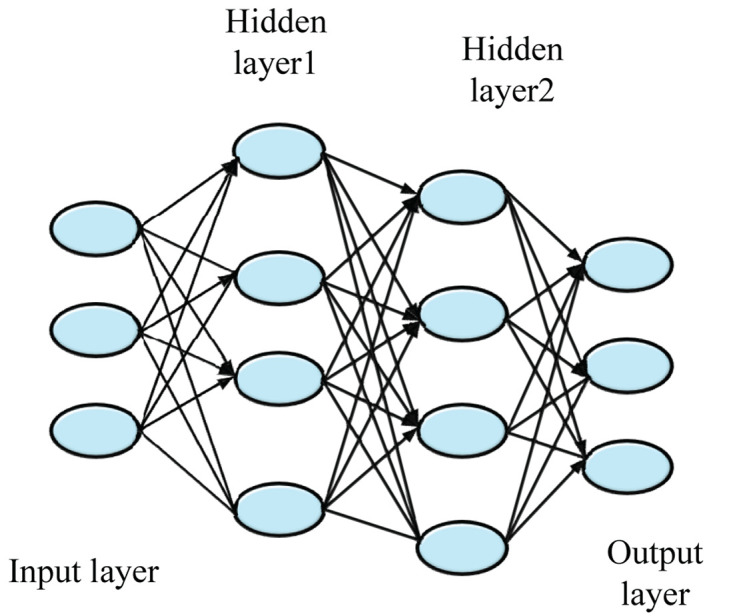
The basic structure of the deep neural network (DNN).

Deep learning technology is a new research direction in the field of ML, which has the ability of autonomous learning and screening information. In the process of learning, the ability of students to analyze and use resources is uneven. This section focuses on how to use the DL algorithm to recommend reliable and effective learning resources for unique situations of students. The artificial neural network (ANN) is an extension of a perceptual network, while DNN is an ANN with many hidden layers (Zhou et al., 2018). According to the location, the ANN layers in DNN can be divided into the input layer, hidden layer, and output layer. Generally, the first layer is the input layer, the last layer is the output layer, and the middle layers are the hidden layer, as shown in Figure 1.

The DNN is considered an ANN with multiple hidden layers, which can deeply excavate data. The basic structure of DNN includes the input layer, hidden layer, and output layer. Layers are fully connected (Qian et al., 2018; Zawacki-Richter et al., 2019), that is, any node of layer *i* is connected to any node of layer *i* + 1. The input and output layers form a linear relationship, as shown in Eq. (1).


(1) $$z=\sum_{i=1}^{m}w_{i}\,x_{i}+b$$

where, *z* represents the output result, *w* is the linear relationship coefficient, *x* denotes the input variable, and *b* stands for the bias parameter. The expression of the Sigmoid activation function selected to complete the subsequent operation reads as follows:


(2) $$\delta \,\left(z\right)=\frac{1}{1+e^{-z}}$$

where, δ(*z*) is the activation function. The parameters are calculated through backpropagation (BP) and gradient descent. Moreover, the weight in the network is adjusted for faster model performance, which is of great significance to improve the system performance and training speed.

**FIGURE 2:**
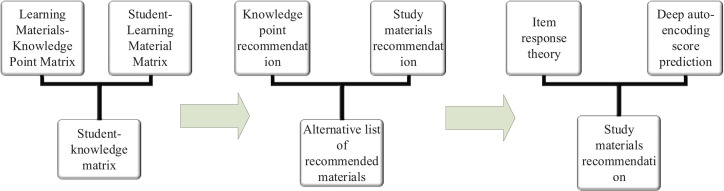
Personalized learning material recommendation algorithm.

In this study, a personalized learning material recommendation algorithm is proposed based on DNN. The algorithm flow is shown in Figure 2.

Figure 2 depicts the main flow of the proposed recommendation method: (1) data set is analyzed and processed, the interactive records between students and learning materials are obtained, and the correlation matrix is constructed between test questions and knowledge points (Im et al., 2018; Chen, 2019). (2) Learning materials are recommended based on secondary collaborative filtering of knowledge points. First, knowledge points are recommended based on the student knowledge point matrix, and then learning materials containing recommended knowledge points are recommended. (3) Based on the item response theory, a prediction model is constructed to judge whether the steps of students using the knowledge point in the recommended learning materials are right. (4) The recommended test questions are predicted based on the scores of students. (5) The final recommended list of learning materials is determined, and the difficulty of learning materials is controlled within a reasonable range.

**FIGURE 3:**
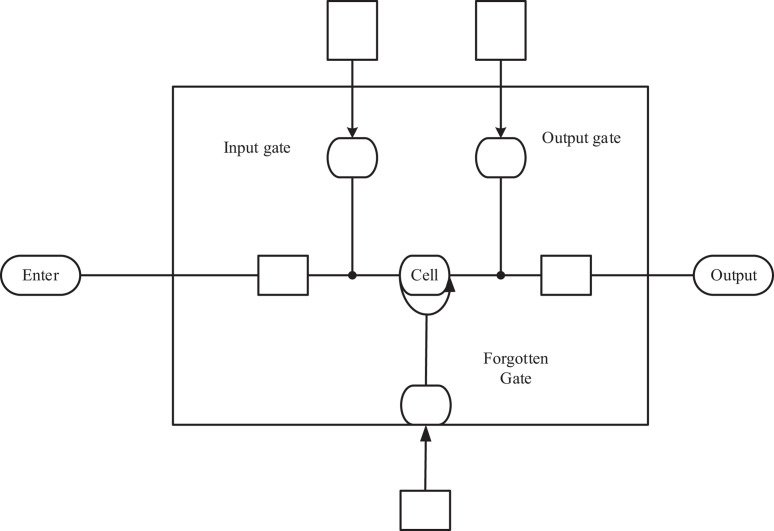
The cell structure of long short-term memory.

Based on the structural principles and early learning theory in Bruner’s structural education theory, the order of learning resources should be considered in learning courses and knowledge points of students. The current learning content is affected by historical learning behaviors, as well as the future learning objectives and learning paths. Therefore, it is recommended to select bi-directional long short-term memory (Bi-LSTM) neural network (NN) that is good at tackling sequence problems in the second test based on knowledge points (Wu and Song, 2019; Tschandl et al., 2020). The unit structure of LSTM is shown in Figure 3.

The long short-term memory unit is composed of unit structures as shown in Figure 3. The unit structure includes three important parts, namely, the forgetting gate, input gate, and output gate (Wu et al., 2019). Forgetting gate can judge which data to be transmitted downward and forget the information that is not to be transmitted downward. The input gate can decide what content is added to the network and send it downward. The output gate can determine the information output to the next cell structure.

**FIGURE 4:**
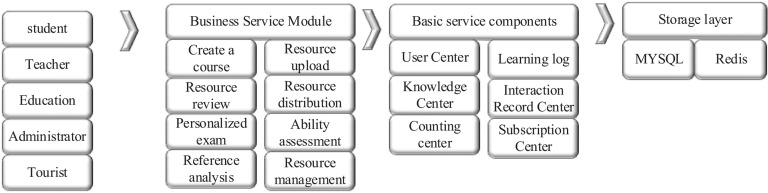
The architecture of an adaptive learning system based on DNN.

Based on the above learning material recommendation algorithm, an adaptive learning system based on DNN is proposed. The basic architecture of the system is shown in Figure 4.

As shown in Figure 4, the adaptive learning system is divided into the online learning subsystem, personalized examination subsystem, learning situation analysis subsystem, and resource management subsystem according to business functions. In the online learning subsystem, students subscribe and learn the course according to their needs. When they reach a certain stage of learning, they can sign up for the examination issued by the course teacher to consolidate the practice. In the personalized examination subsystem, the system provides personalized examination services according to learning records and question-making records of students for targeted improvement. The learning situation analysis subsystem uses the classification of educational objectives to diagnose and analyze the cognition of students (Hu et al., 2020). In the resource management subsystem, the teacher user manages the uploaded curriculum resources, and the educational administration user manages the curriculum, users, and announcements within the management scope.

### Simulation and Experimental Design

The experimental design focuses on the processing and analysis of knowledge points. On the one hand, through the information extraction of each question record, the correct and wrong information of corresponding knowledge points and steps on specific questions of students can be obtained. Then, statistical analysis is conducted based on knowledge points to obtain mastery of knowledge points of students (i.e., accuracy). Finally, the last interaction record time between students and the knowledge point is used as the timestamp information, so that the information of students and test questions, knowledge points, cognition, and time matrix can be obtained. On the other hand, through the knowledge-based processing of the overall data set, the relevant information of the knowledge point is obtained. For example, the importance of the knowledge point is expressed by the number of occurrences of the knowledge point, and the difficulty of the knowledge point is expressed by the error rate of the student group on the knowledge point.

The experimental evaluation standard adopts the precision, recall, *F* value (F1), and average difficulty (AD) as the evaluation indexes, and the calculation equation reads as follows:


(3) $$p\,r\,e\,c\,i\,s\,i\,o\,n=\frac{H\,i\,t}{R}$$

(4) $$r\,e\,c\,a\,l\,l=\frac{H\,i\,t}{T}$$

(5) $$A\,D=\frac{\sum_{\text{i}=1}^{\text{n}}D}{n}$$

In Eqs. (3)–(5), *Hit* represents the number of questions the user has practiced, *R* indicates the total number of recommended questions, *T* denotes the actual total number of questions, *D* means the difficulty of each question, and *n* is the number of questions.

The data set used in the experiment is the data set of the test results of law majors in some universities in China, as well as the interactive log between the intelligent teaching guidance system and students. In addition, the data set contains 18,000 records of more than 300 students, each of which is detailed information of the performance of students in the practice. The content of the personalized recommendation system is to understand cognition of language points, important and difficult points, and other related information of students. The data set is processed and analyzed according to language points according to the characteristics of the data set and the needs of the personalized recommendation algorithm based on DL. Through the information extraction of each project, the information of the language point on the specific questions in the test is obtained. After the language points are analyzed, mastery of the language points of students is obtained. Finally, the last interactive time between students and their mastery of the language point is seen as the time information, which can help to obtain the matrix of students and questions, language points, cognition, and time matrix. After the data set based on language points is processed, the information of the language points can be obtained. For example, the number of language points indicates the importance of the language point, and the error rate of mastery of the language points of students indicates the difficulty of the language point.

## System Performance Test Results and Discussion

### Model Parameter Adjustment Results

**FIGURE 5:**
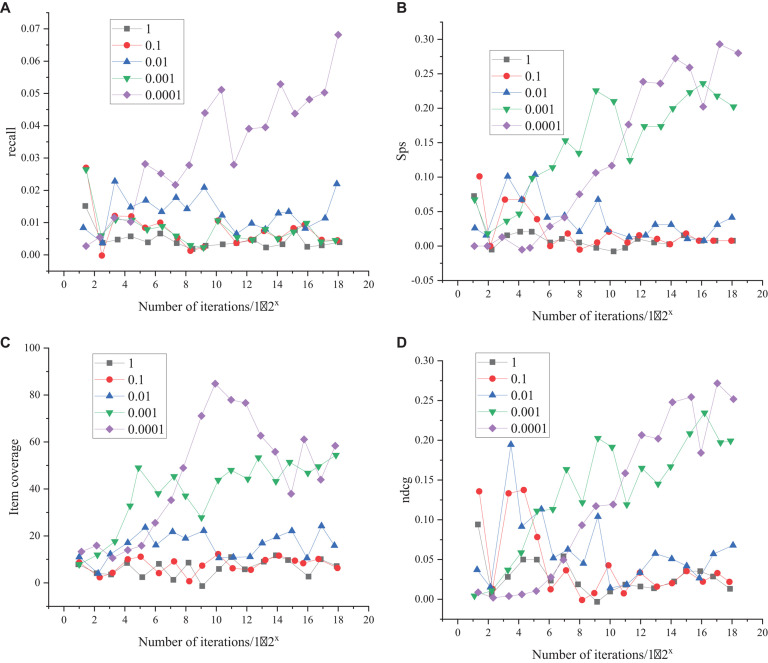
Learning rate parameter selection results **(A)** represents the comparison results of recall under different learning rates, **(B)** indicates the comparison results of SPS values under different learning rates, **(C)** denotes the comparison results of item coverage under different learning rates, and **(D)** is the comparison results of item recommendation quality under different learning rates, represented by ncdg.

Before the recommendation system verification, the NN parameters should be adjusted to determine the model structure. The adjustment results of learning rate parameters of the Bi-LSTM NN are shown in Figure 5.

As shown in Figure 5, the most suitable learning rate is selected from 1, 0.1, 0.01, 0.001, and 0.0001 in turn for comparative experiments. In terms of the recall, Figure 5A suggests that when the learning rate is 0.0001, the recall of the training model has obvious advantages; with the increase of training iterations, the advantage of model recall becomes more obvious. In terms of the other three indicators, although the growth trend of each indicator is the same when the learning rate is 0.0001, the growth of each indicator is more advantageous when the learning rate is 0.0001. Therefore, it is determined that the best learning rate of model training is 0.0001.

**FIGURE 6:**
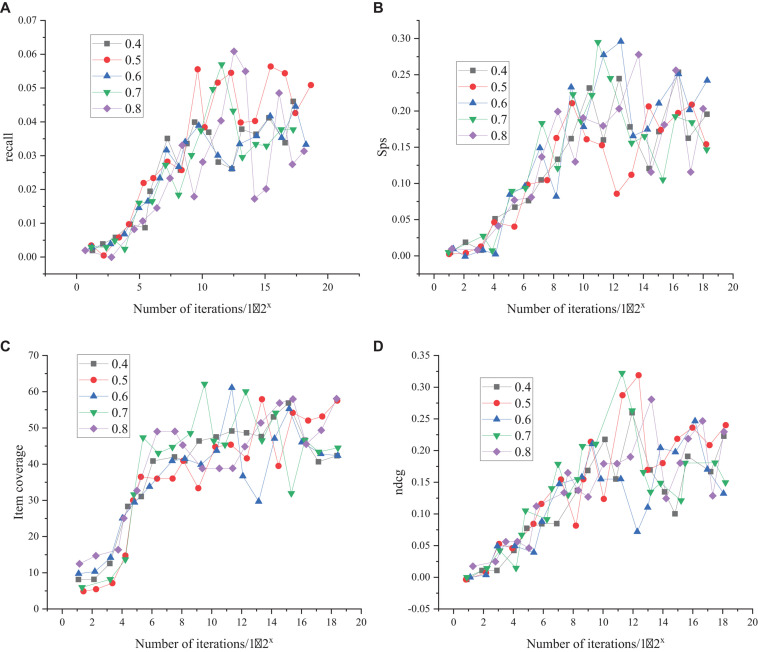
Dropout parameter selection results **(A)** represents the comparison results of recall under different dropouts, **(B)** denotes the comparison results of SPS values under different Dropouts, **(C)** indicates the comparison results of item coverage under different Dropouts, and **(D)** is the comparison results of item recommended quality under different dropouts, represented by ncdg.

The selection and comparison results of dropout parameters of the training model are shown in Figure 6.

As shown in Figure 6, with the progress of training, when Dropout is 0.4, 0.5, 0.6, 0.7, and 0.8, there is a similar changing trend in the recall, SPS value, item coverage, and item recommendation quality. However, the changing trend is unstable when Dropout is 0.7 and 0.8, while it is stable in the other three cases. Overall, the Dropout value is 0.5.

**FIGURE 7:**
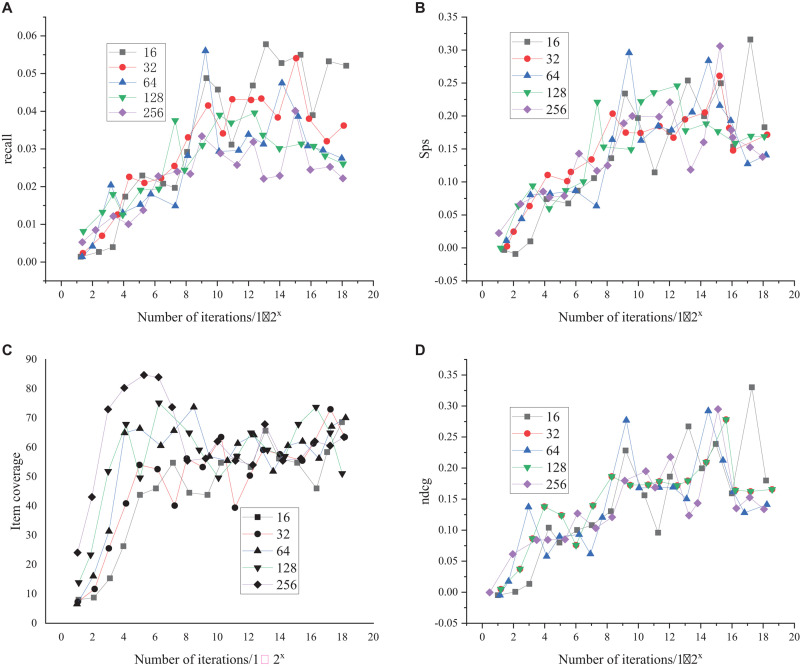
Selection results of batch size parameters of the training model **(A)** represent the comparison results of recall rates under different batch sizes, **(B)** denotes the comparison results of SPS values under different batch sizes, **(C)** indicates the comparison results of item coverage under different batch sizes, and **(D)** is the comparison results of item recommendation quality under different batch sizes, represented by ncdg.

The comparison of the selection results of the batch size parameter of the training model is shown in Figure 7.

As shown in Figure 7, as the number of training iterations increases, when the batch size parameters are 16, 32, 64, 128, and 256, the changing trends of recall, SPS value, and project recommendation quality are similar. When the batch size is 256, the item coverage performs well at the beginning of training and then tends to be stable. When the batch size is 16 and 64, the performance of the four groups of indicators fluctuates. Based on the results of each parameter and its stability, the batch size is finally determined as 32.

### Comparison Results of System Performance Verification

**FIGURE 8:**
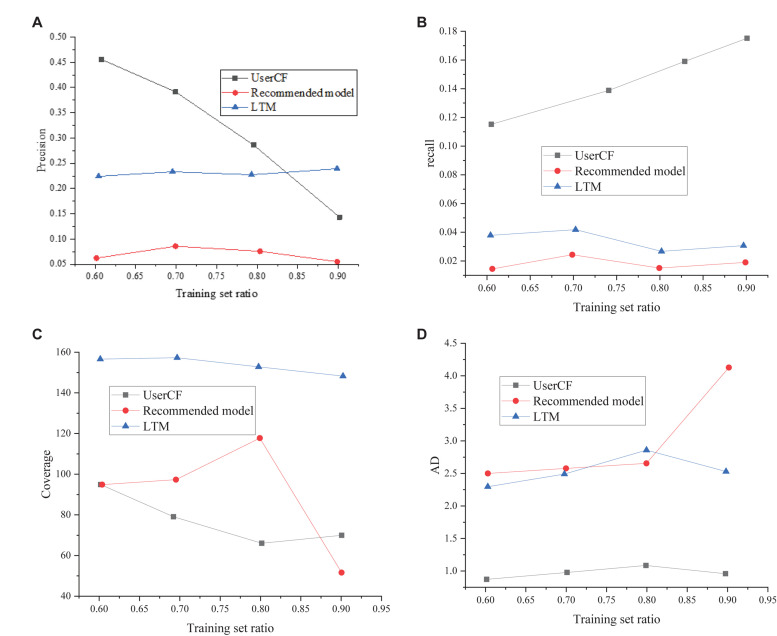
Performance verification and comparison results. **(A)** The accuracy comparison result; **(B)** the recall ratio (recall) comparison result; **(C)** the coverage ratio (Coverage) comparison result; **(D)** the average difficulty (AD) comparison result.

After the training, ANN parameters are determined, the model performance is verified, and the User-based Collaborative Filtering (UserCF) model and lifelong topic modeling (LTM) are selected as the control groups. The comparison results are shown in Figure 8. UserCF is one of the most classic and widely used recommendation algorithms, and its recommendation process is divided into two stages. First, the users with similar interests or usage experiences are searched out to form the user groups, and then, the items that the users never used are recommended. The LTM recommendation algorithm is an effective method to introduce the time element into the recommendation method. The word2vec algorithm is first used to include the elements used by users in the training set, and then, the optimal interpretable translation vector is estimated for the elements contained by each user. Finally, the prediction of the last translation vector of the user is obtained, and some suggestions are proposed.

Figure 8 presents that with the increase of the training set, the accuracy of the traditional UserCF begins to decline significantly, while the accuracy of the LTM remains at a low level. The proposed method is more stable, and the model performance has been improved. In terms of the recall, the recall of the traditional UserCF method fluctuates obviously with the increase of the proportion of the training set. Although the recall of the UserCF method and LTM is low, the value has remained stable. The difficulty of the test question resources recommended by this method is relatively stable, and full consideration is given to whether the investigation on the recommended knowledge points on the recommended test questions is too difficult for the target students, which has good interpretability. However, the difficulty of recommended test questions by traditional methods is generally low and does not have a good practice. Based on the above analysis, the personalized learning resource recommendation method based on DL proposed still has good stability under the conditions of various training data scales. The recommended personalized test questions of students are moderately difficult. It is easier to interpret the recommendation according to the acquisition of knowledge points of students and whether the recommended test questions meet the practical objectives of students.

The above results show that the recommendation accuracy of the traditional UserCF and the LTM method is low, while that of the proposed method is stable and improved. The comparison results of recall show that the recall value of UserCF fluctuates significantly with the increase of the training set. Although the recall value of the proposed method and LTM is low, the value remains stable. The difficulty of the materials recommended by the method is stable, and it focuses on whether the investigation of the recommended learning materials is difficult for the target students. However, the difficulty of learning materials recommended by UserCF is not high and practical, and those recommended by LTM are not stable, either. Based on the above analysis, it is concluded that the personalized recommendation method of learning materials based on DL is stable no matter how big the size of the sample is. The difficulty of the exercises recommended by the personalized recommendation is moderate, and it forces on the mastery of the language points of students and whether the recommendation test can help students understand the language point.

## Conclusion

This study first analyzes the relevant research of AI algorithms in the field of education and summarizes the advantages and disadvantages of the basic recommendation algorithms from the perspective of educational psychology, through the combined application of AI and DL algorithms in legal education. The related theories of pedagogy are introduced. Combined with the DL algorithm, a personalized learning material recommendation algorithm based on DNN is proposed, and a Bi-LSTM algorithm is introduced for optimization. Thereon, an adaptive learning system architecture based on DNN is proposed. The results show that the personalized learning resource recommendation method based on DL proposed still has good stability under various training data scales. The personalized test questions of recommended students are moderately difficult. According to the acquisition of knowledge points of students and whether the recommended test questions meet the practical objectives of students, the proposed method can easily interpret the recommendation, and meet the personalized needs of legal education. Although the research has achieved some results, there are still some limitations. First, due to the limited research funds and research level, the research and design field is not wide, which brings some errors to the results. Second, the research on DNN is still in the shallow layer. In future research, the above two points will be improved to make the research results more accurate and improve the persuasion of this study.

## Data Availability Statement

The raw data supporting the conclusions of this article will be made available by the authors, without undue reservation.

## Ethics Statement

The studies involving human participants were reviewed and approved by Changzhou University and Shanghai Jiao Tong University Ethics Committee. The patients/participants provided their written informed consent to participate in this study. Written informed consent was obtained from the individual(s) for the publication of any potentially identifiable images or data included in this article.

## Author Contributions

All authors listed have made a substantial, direct and intellectual contribution to the work, and approved it for publication.

## Conflict of Interest

The authors declare that the research was conducted in the absence of any commercial or financial relationships that could be construed as a potential conflict of interest.

## Publisher’s Note

All claims expressed in this article are solely those of the authors and do not necessarily represent those of their affiliated organizations, or those of the publisher, the editors and the reviewers. Any product that may be evaluated in this article, or claim that may be made by its manufacturer, is not guaranteed or endorsed by the publisher.
